# Emerging Roles of the Iron Chelators in Inflammation

**DOI:** 10.3390/ijms23147977

**Published:** 2022-07-20

**Authors:** Alessandra Di Paola, Chiara Tortora, Maura Argenziano, Maria Maddalena Marrapodi, Francesca Rossi

**Affiliations:** Department of Woman, Child and General and Specialist Surgery, University of Campania “Luigi Vanvitelli”, Via Luigi De Crecchio 4, 80138 Naples, Italy; alessandra.dipaola@unicampania.it (A.D.P.); chiara.tortora@unicampania.it (C.T.); maura.argenziano@unicampania.it (M.A.); mariamaddalena.marrapodi@studenti.unicampania.it (M.M.M.)

**Keywords:** iron, inflammation, iron chelation, anti-inflammatory properties, deferiprone, deferoxamine, deferasirox, Dp44mT, eltrombopag

## Abstract

Iron is a crucial element for mammalian cells, considering its intervention in several physiologic processes. Its homeostasis is finely regulated, and its alteration could be responsible for the onset of several disorders. Iron is closely related to inflammation; indeed, during inflammation high levels of interleukin-6 cause an increased production of hepcidin which induces a degradation of ferroportin. Ferroportin degradation leads to decreased iron efflux that culminates in elevated intracellular iron concentration and consequently iron toxicity in cells and tissues. Therefore, iron chelation could be considered a novel and useful therapeutic strategy in order to counteract the inflammation in several autoimmune and inflammatory diseases. Several iron chelators are already known to have anti-inflammatory effects, among them deferiprone, deferoxamine, deferasirox, and Dp44mT are noteworthy. Recently, eltrombopag has been reported to have an important role in reducing inflammation, acting both directly by chelating iron, and indirectly by modulating iron efflux. This review offers an overview of the possible novel biological effects of the iron chelators in inflammation, suggesting them as novel anti-inflammatory molecules.

## 1. Introduction

Iron is an important element involved in different physiologic and pathologic mechanisms in mammalian cells [1]. Its body content varies according to sex, health and nutrition and is about 3–5 g in adults. It could be present in two forms into cells, ferrous (Fe^2+^) and ferric (Fe^3+^), and it is a constituent of several metalloproteins, for example in heme as organic cofactor, and in different function groups as an inorganic cofactor, such as iron-sulfur clusters [2].

Iron participates to cellular metabolism, DNA synthesis and repair, cell growth and death [3]. Although iron is important for life processes, its excess is highly toxic because it activates the Fenton reaction, during which iron reacts with hydrogen peroxide and generates hydroxyl radicals. These toxic free radicals are responsible for cellular proteins, lipids, and nucleic acids damage [3,4]. Moreover, iron homeostasis is also involved in the regulation of immune system by modulating the activities of innate and adaptive immunity cells, among them T and B cells, neutrophils, macrophages [1]. For these reasons its metabolism is finely regulated, and its alteration is responsible for the onset of several diseases [1]. In particular, several iron-processing cells and tissues are involved in regulation of iron homeostasis and also the hepcidin/ferroportin axis plays a crucial role in this regulation. Alterations of iron import and export can determine the onset of different disorders [3].

There are several biomolecules which modulate iron homeostasis, contributing to its import and export in cells, and also to its peripheral transport to target cells [1,5].

**Figure 1:**
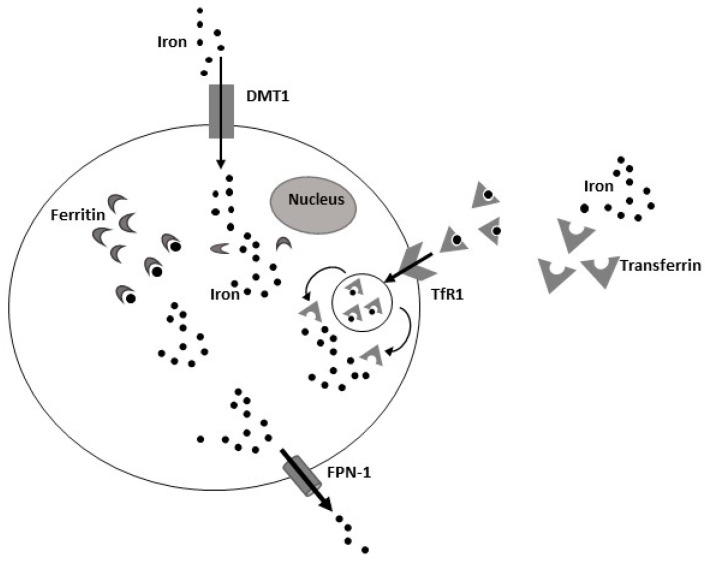
Iron metabolism. Several biomolecules modulate iron metabolism, contributing to its import and export in cells. Transferrin (Tf) is an important glycoprotein responsible for the transport of circulating iron in its inert state. The Transferrin Receptor-1 (TfR-1) and the Divalent Metal Transporter 1 (DMT1) are the main iron importers, located on cell surface, responsible for iron internalization in cells. TfR-1 recognizes and internalizes the complex Tf-ferric iron (Fe^3+^) into cells, where iron is released, while DMT1 binds and internalizes reduced iron (Fe^2+^). Once iron has entered the cell, it can be used by the mitochondrion for metabolic reactions, or it can be bound in its inert form by ferritin. Then, excess iron is released by cell through the only known iron efflux protein ferroportin-1 (FPN-1).

Transferrin (Tf) is an important glycoprotein responsible for the transport of circulating iron in its inert state [6]. The Transferrin Receptor-1 (TfR-1) and the Divalent Metal Transporter 1 (DMT1) are the main iron importers, located on cell surface, responsible for iron internalization in cells. TfR-1 recognizes and internalizes the complex Tf-ferric iron (Fe^3+^) into cells, where iron is released [6,7], while DMT1 binds and internalizes reduced iron (Fe^2+^). Once iron has entered the cell, it can be used by the mitochondrion for metabolic reactions or it can be bound in its inert form by ferritin, the main iron storage protein capable of binding up to 4500 atoms of iron [6,8]. Then, excess iron is released by the cell through the only known iron efflux protein ferroportin 1 (FPN1) [8,9] (Figure 1).

In conclusion, iron metabolism is finely regulated so that its intracellular and circulating concentration falls within the physiological ranges. Otherwise, dysregulation of iron metabolism can induce an increase of intracellular iron concentration which plays a key role in inflammatory processes, and is the cause of the onset of several inflammatory and immune diseases [10,11,12].

## 2. Iron Homeostasis Alteration in Inflammation

Iron homeostasis can be regulated at two different levels: cellular and systemic levels [8]. Iron regulatory proteins (IRPs), IRP1 and IRP2, are involved in the regulation of iron homeostasis at cellular levels, by modulating the levels of DMT1, TfR-1, FPN1 and ferritin [8]. When there are low levels of iron in cells, IRP1 and IRP2 recognize and bind the iron responsive elements (IREs) located in the untranslated regions (UTRs) of the messenger RNAs (mRNAs) encoding various proteins involved in iron metabolism, modulating their translation [5,13]. Conversely, when iron levels are high in cells, IRPs are not able to bind IREs in UTR of these mRNAs [5,13].

The peptide hormone hepcidin is mainly responsible for the regulation of iron homeostasis at a systemic level. It is an 84-amino-acid long prepropeptide produced by hepatocytes after different stimuli: when iron concentration reaches high levels in serum, during systemic inflammation (in particular, when interleukin (IL)-6 levels are increased) or during hypoxia [8,14,15]. Pre-prohepcidin is cleaved at level of 24-amino-acid N-terminal signal peptide to obtain prohepcidin, constituted by 60 amino acids. Mature hepcidin (25-amino-acid form) is obtained after furin-like prohormone convertases cleavage at level of pro-region [14]. Mature hepcidin includes:

- A C-terminus characterized by four highly conserved disulphide bonds, which creates a β-hairpin; and
- An unstructured N-terminus essential for FPN1 interaction [14].

The interaction between hepcidin and FPN1 plays a key role in regulation of iron homeostasis [14]. In particular, Hepcidin causes FPN1 degradation, by binding, internalizing it into cell, and eventually degrading it, thus inhibiting iron release by cells and contributing to its accumulation in cells [14,15,16].

Hepcidin expression is mainly regulated by the bone morphogenetic protein/Sma mothers against the decapentaplegic (BMP/SMAD) pathway [14]. The ligand BMP6 is considered the main BMP responsible for hepcidin transcription [17]. More in detail, high levels of body iron stimulate liver BMP6 expression, which recognizes membrane-bound BMP receptors (BMPRs), binding it together with hemojuvelin [14]. After the binding between BMP6 and BMPRs, a phosphorylation of intracellular SMAD1/5/8 occurs, and the phosphorylated form migrates into the nucleus and binds to the hepcidin gene promoter at level of BMP-responsive elements (BMP-RE), promoting hepcidin expression [14].

**Figure 2:**
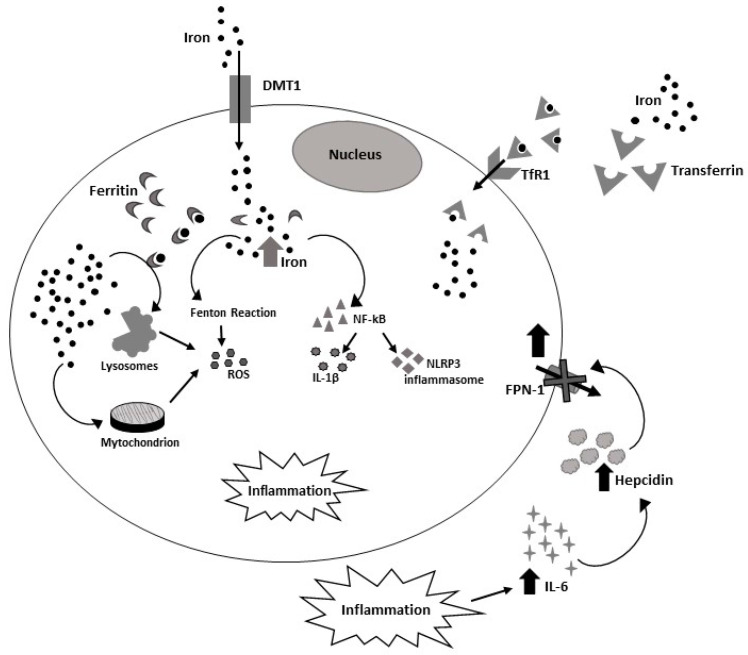
Iron homeostasis alteration in inflammation. During inflammation high levels of interleukin-6 (IL-6) cause an overexpression of Hepcidin responsible for ferroportin (FPN1) degradation and consequently for intracellular iron accumulation. Free iron accumulation is highly toxic for cells, causing reactive oxygen species (ROS) production which causes damage of mitochondria, lysosomes, DNA, proteins and lipids causing senescence, cell death, and inflammation. Oxidative stress increases the level of pro-inflammatory cytokines and also upregulates nuclear factor-kappa B (NF-κB) leading to an increase of the inflammatory state responsible for the onset of several chronic diseases.

An alteration of the hepcidin-FPN1 axis is responsible for the onset of several iron disorders. Increased levels of hepcidin inhibit iron release by cells resulting in a reduction of circulating iron levels. Conversely, the reduction of hepcidin levels determines a major iron release by cells causing an iron overload [18]. Hepcidin levels are closely dependent on inflammation, in particular on IL-6 levels. Indeed, during acute and chronic inflammation, high levels of IL-6 determine an increase in hepcidin levels which are not only responsible for reduction of circulating iron, but also of iron accumulation in cells which are unable to release it because of FPN1 degradation by hepcidin [19,20]. Free iron accumulation is highly toxic for cells given its capability to both accept and release electrons switching between the Fe^2+^ and Fe^3+^ forms and participating in the generation of reactive free radicals in aerobic organisms [10,12]. In particular, the cellular labile iron pool (LIP), constituted by redox-active iron (Fe^2+^), is responsible for production of reactive oxygen species (ROS) which cause damage to DNA, proteins and lipids causing senescence, cell death, inflammation, and leading to the onset of several diseases [11]. Excessive ROS production is involved in inflammatory processes by inducing an increase in pro-inflammatory cytokine production and release [21]. Indeed, it has been reported that oxidative stress increases the level of pro-inflammatory cytokines such as tumor necrosis factor-α (TNF-α) and IL-6 and also upregulates nuclear factor-kappa B (NF-κB), leading to an increase of the inflammatory state responsible for the onset of several inflammatory and autoimmune diseases [21,22] (Figure 2). Therefore, the alteration of iron metabolism causes an impairment of cell metabolism, by promoting ROS accumulation. In turn, ROS, produced by the electron transport chain, could be involved in Fe^2+^ and Fe^3+^ release, causing mitochondrial stress [23]. Mitochondrion is the main target of iron-mediated oxidative stress [24]. Indeed, mitochondria functions are principally dependent on iron uptake, using it for formation of iron-sulfur clusters, heme synthesis, and its storage in mitochondrial ferritin [25]. Therefore, the impairment of iron homeostasis or mitochondrial iron metabolism could induce cellular stress, causing an inflammatory state, which is the basis of several autoimmune and inflammatory diseases [26]. In particular, it has been demonstrated that the mitochondria impairment caused by the alteration of iron homeostasis is implicated into pathogenesis of systemic lupus erythematosus (SLE), leading to a chronic inflammatory state [23]. Moreover, in coronavirus disease 2019 (COVID-19), a severe systemic inflammatory disease [27], a lot of complications are closely related to intracellular iron accumulation which is consequently responsible for mitochondria function alteration, determining free radicals, ROS, and pro-inflammatory factors release [28]. Another subcellular organelle damaged by iron metabolism impairment is represented by the lysosome. It is an acidic digestive organelle in which most of the iron recycling occurs [29]. An anomalous absorption of iron could worsen oxidative tissue damage in different inflammatory lung disorders [30]. In particular, the redox-active iron contained in the lysosomes is the main responsible for these organelles’ impairment due to oxidative stress. Interestingly, it has been demonstrated that lysosomal iron chelation in respiratory epithelial cells avoids lysosomal damage and, consequently, cell death [30]. An alteration of iron metabolism is reported in Inflammatory Bowel Diseases (IBD), Crohn’s disease (CD), ulcerative colitis (UC), which are chronic inflammatory disorders of the gastrointestinal tract [31]. It has been revealed that iron accumulation alters gut microbial homoeostasis, worsening intestinal inflammation both in a murine model and in a DSS-induced colitis rat model [32]. An excess of ROS production is reported in UC and, interestingly, it has been demonstrated that the administration of iron induces a decrease of ROS production, reducing colonic symptoms in IBD [32]. Xu and collaborators proposed a relationship between IBD and ferroptosis observing that high levels of iron in the intestine are responsible for ROS generation, lipid peroxidation, oxidative stress, and cell death [32]. Ferroptosis is reported to be involve in both clinical UC patients and in murine experimental colitis [33,34]. Ferroptosis is a type of regulated cell death dependent on iron concentration, whose accumulation causes lipid damage [35]. It is characterized by an iron-related peroxidation of phospholipid membranes rich in polyunsaturated fatty acids (PUFAs), which determines cell death [36]. Iron is involved in lipid peroxide accumulation and, consequently, in ferroptosis. Therefore, iron metabolism is closely responsible for ferroptosis regulation [35]. Dixon and collaborators in 2012 first introduced the term ferroptosis, referring to a newly identified mechanism of programmed cell death mediated by iron-dependent lipid peroxidation of cell membranes and distinct from other known forms of programmed cell death [37]. The events triggering ferroptosis are erastin-mediated glutathione (GSH) depletion and phospholipid peroxidase glutathione peroxidase 4 (GPX4) inactivation [37,38]. GPX4 is a crucial molecule responsible for conversion of potentially toxic lipid hydroperoxides in non-toxic lipid alcohols, therefore its inactivation causes lipid peroxidation responsible for cell death [35]. Mitochondrial voltage-dependent anion channels (VDACs) play an important role in ferroptosis. Indeed, the opening of these channels caused by erastin induces iron entry into the mitochondria, ROS production, and, consequently, an increase of both mitochondrial potential and oxidative stress, processes involved in ferroptosis [39]. Ferroptosis-related cell death could be inhibited by the depletion of PUFAs, and the administration of lipid peroxidation inhibitors, lipophilic antioxidant, and iron chelators [35]. In recent years, it has been demonstrated that there is a relation between ferroptosis and inflammation. Several pro-inflammatory cytokines are involved in ferroptosis by regulation of GPX4 levels and activity in cancer cells [40,41]. A strong downregulation of GPX4 was observed in these cells treated with TNF, inducing ferroptosis initiation [42]. Moreover, it is reported that in mice with hemochromatosis the pro-inflammatory IL-6/hepcidin signaling has a crucial role in promoting ferroptosis and that the administration of the anti-inflammatory drug auranofin could inhibit ferroptosis [40]. Considering the involvement of iron in inflammation and the relation between inflammation and ferroptosis, chelating iron could be considered an interesting therapeutic approach to reduce the inflammatory state, which is particularly compromised in several autoimmune and inflammatory disorders. These drugs could act directly on inflammation by chelating iron ions, and also indirectly by counteracting ferroptosis.

IBD chronic inflammation is also responsible for the onset of osteoporosis (OP) in these patients. In particular, high levels of pro-inflammatory cytokines in CD and UC alter bone metabolism inducing bone resorption by determining an increase of the ratio of receptor activator of NF-κB ligand (RANK-L)/Osteoprotegerin (OPG) [31,43]. It has been demonstrated that an alteration of iron metabolism is involved in IBD pathogenesis, with an accumulation of intracellular iron and a reduction of circulating iron [31,44]. The dysregulation of iron metabolism in IBD depends on the overactivation and production of hepcidin, derived by the increased levels of inflammatory state in IBD and, in particular, by high concentration of IL-6 [31,35,39].

Also in celiac disease, an autoimmune disorder, an alteration of iron metabolism caused by its impaired inflammatory state is reported [45]. A prevalence of M1 pro-inflammatory macrophages together with an excess of intracellular iron concentration could be involved in celiac disease pathogenesis, contributing to inflammation [45].

Inflammation-related iron accumulation and the consequent ROS production are also involved in the pathogenesis of inflammatory skin disease [46]. Indeed, skin is very susceptible to these inflammatory stimuli due to its polyunsaturated fatty acids composition and its exposure to ultraviolet light, which participate in ROS production [46]. Interestingly, it has been reported that iron accumulation in macrophages is responsible for an excessive stimulation of pro-inflammatory macrophages in chronic venous disease (CVD) [47]. Moreover, intracellular iron accumulation in macrophages, due to alteration of hepcidin/FPN1 signaling alteration, is also responsible for impairment of wound healing [47].

It is known that iron overload is responsible for osteoclast (OC) overactivation, contributing to bone resorption [48]. Rossi et al. demonstrated that iron overload induced an increased expression of OC marker tartrate-resistant acid phosphatase (TRAP), determining OC overactivity, bone resorption, and consequently OP onset [36].

Iron overload is also responsible for development of atherosclerosis. Indeed, hepcidin inhibition during early- to mid-stage plaques reduced pro-inflammatory macrophages activities [3].

Since iron is involved in inflammatory conditions, targeting iron metabolism and chelating iron could be considered a novel potential approach in order to counteract inflammation in several disease. This review offers an overview of the possible novel biological effects of the iron chelators in inflammation, suggesting them as novel anti-inflammatory drugs.

## 3. Iron Chelators as Anti-Inflammatory Drugs

The accumulation of iron due to its impaired homeostasis can be particularly dangerous. The iron toxicity is caused by its potential to induce oxidative stress thought ROS production and consequently inflammation [49]. ROS can affect DNA, protein and lipid integrity damaging cellular functionality [50]. Iron-mediated inflammation depends on the pro-inflammatory nature of the excessive free iron and of the iron bound to the storage protein, ferritin [51,52]. The cellular free iron promotes inflammation through the NF-κB pathway inducing IL-1β secretion and NLRP3 inflammasome stimulation in human monocytes [51]. Equally, ferritin acts as a local cytokine inducing an increase of pro-inflammatory cytokines such as IL-1β [52]. Moreover, inflammation itself induces iron accumulatio in tissues such as liver upregulating hepcidin [53] and thus reducing FPN1 causing the establishment of a vicious circle, where iron-mediated inflammation generates in turn an increase of intracellular iron levels which consequently induces oxidative stress and iron-mediated inflammation [49].

A multivariate analysis revealed a strong association between high levels of iron in the blood and reduced health span [54]. Iron-mediated oxidative stress induces inflammation and mitochondrial dysfunction playing a critical role in the progression of several inflammatory diseases [55]. Immune cells, such as macrophages, developed mechanisms to reduce iron availability during inflammation [1]. In healthy conditions, macrophages degrade hemoproteins and export iron, while during inflammation they retain cytoplasmic iron, reducing extracellular iron concentration. However, at the same time, iron-rich macrophages have a strong inflammatory capability, which contributes to the chronic inflammatory state typical of iron overload conditions [56]. Iron overload is an inevitable consequence of blood transfusions in patients affected by haemoglobinopathies. Therefore, the iron chelation therapy (ICT) is necessary to prevent the consequences of hemosiderosis in these patients and to restore the body iron content [57]. Currently, three iron chelators, approved by the Food and Drug Administration to prevent iron accumulation in patients affected by haemoglobinopathies [57], are available: deferoxamine (DFO), deferiprone (DFP) and deferasirox (DFX). These chelators differ in molecular weight and in intestinal absorption profile [58]. DFO binds to iron in a 1:1 ratio and is administered subcutaneously or intravenously. DFP bind to iron in a 3:1 ratio and it was the first oral chelator proposed. DFX forms complexes with iron in the ratio of 2:1 and it is administered once-daily, offering an important advantage for compliance when compared to the parenterally administered DFO and the thrice-daily orally administered DFP [59,60]. Moreover, they also differ in mechanism of action. DFP and DFX exert their action by targeting cytosolic iron to prevent its incorporation into ferritin, DFO induces ferritin entrance into lysosomes [61,62,63]. Iron homeostasis strongly affects bone metabolism [64]. Several diseases such as hemochromatosis, hemosiderosis, β-thalassemia, sickle cell disease and liver diseases characterized by iron overload are frequently accompanied by OP [65,66]. Iron overload directly promotes bone resorption and inhibits bone formation inducing osteopenia and OP [67]. Excess iron increases osteoclastogenesis and bone resorption [68] while leading to a significant reduction in osteoblast differentiation [69]. Bone loss caused by iron accumulation is also associated with the apoptosis of Bone Marrow Mesenchymal Stem Cells (BM-MSCs) capable of differentiating into osteoblasts [70]. Therefore, the reduction of iron has been suggested as a potential therapeutic approach for OP treatment. In 2014, Rossi et al. demonstrated that iron overload induces osteoclast (OC) overactivation in β Thalassemia Major patients and that OCs activity can be reduced with a chelation therapy. They tested the effects of DFO, DFP and DFX demonstrating the stronger effect of DFX in reducing OCs activity [36]. A few years later, Punzo et al. tested Eltrombopag (ELT), an agonist at Thrombopoietin receptor with chelating properties, in combination with DFX in iron overloaded OCs from thalassemic patients demonstrating that also ELT is able to reduce bone mass loss [71]. Recently, also the iron chelator di-2-pyridylketone-4,4-dimethyl-3-thiosemicarbazone (Dp44mT) has been proposed as an anti-inflammatory drug [72]. The role of iron chelators as possible anti-inflammatory agents has been investigated in both in vitro and in vivo experiments as reported in the following paragraphs. The studies reported provide insight into the potential anti-inflammatory protective effects of these iron chelators, in several inflammatory conditions. However, further studies on the safety and efficacy of the chelators are needed for the clinical application.

### 3.1. Deferoxamine and Deferiprone

DFO and DFP anti-inflammatory properties have been investigated in several inflammatory conditions. Recently, it has been demonstrated the capability of DFO to modulate inflammation in a common inflammatory disease, osteoarthritis (OA) by reducing chondrocyte inflammation and matrix destruction both in vitro and in vivo [73]. A specific relationship between chondrocyte ferroptosis and OA has been proposed. Ferroptosis occurred in chondrocytes induced by IL-1β that is known to induce inflammation and chondrocyte destruction. Guo et al. demonstrated that DFO exerts a protective effect on chondrocytes and slows OA progression by inhibiting chondrocyte ferroptosis [73]. It is known that alteration of iron homeostasis contributes to the pathophysiology of obesity and insulin resistance. Circulating markers of iron overload are positively associated with visceral and subcutaneous fat depots [74]. Moreover, elevated iron concentrations in the body predispose to obesity-associated comorbidities [75,76]. Therefore, the removal of iron excess in obese patients could be an important therapeutic strategy for the management of obesity and the associated comorbidities. The effects of iron chelators have been amply studied in adipose tissue. Wang et al. investigated the anti-inflammatory effect of DFO on lipopolysaccharide (LPS)-induced inflammatory responses in RAW264.7 macrophage cells. They proved that the production of TNF-α, IL-1β, nitric oxide (NO) and prostaglandin E2 (PGE2) induced by LPS and the activation of mitogen-activated protein kinases (MAPKs) and NF-κB signaling pathways was significantly inhibited by DFO administration [77]. In agreement, recently, Yan et al. demonstrated that DFO reduces inflammatory marker (TNFα, IL-2, IL-6, and Hepcidin) secretion and the hypertrophic adipocytes size of ob/ob mice [78]. Moreover, an in vitro study proved that DFO is able to inhibit IL-6 induced by iron in human pre-adipocytes but it is not able to alter the adiponectin release [79]. It has been hypothesized that iron accumulation in the liver can induce the progression of steatosis to the next phase of NAFLD such as steatohepatitis, fibrosis and cirrhosis [80]. Xue et al. observed that DFO reduces hepatic cell apoptosis modulating Bcl-2, Bax and cleaved caspase-3 proteins expression, hepatic inflammatory markers (IL-1β, IL-2, and IL-6) and also oxidative stress [81]. In agreement, Mohammed et al. demonstrated an antifibrotic effect for DFO in acute hepatotoxicity. They proved that DFO reduces lipid peroxidation, increased SOD and glutathione peroxidase and reduced fibrosis markers and the activation of the stellate cells which produce tumor growth factor-β (TGF-β) [82]. As regards DFP, Zou et al. demonstrated its protective effects in Diabetic cardiomyopathy (DC), a chronic and low-level inflammation disease, by the regulation of inflammatory signal pathways [83]. In particular, they demonstrated that DFP, reduces the levels of inflammatory and fibrosis relevant factors, such as NF-kB, COX2, tenascin C and collagen IV exerting an important protective effect on myocardial damage in DC rats [83]. Recently, Ramezanpour et al. evaluated the potential anti-inflammatory effect of DFP suggesting it as a possible compound to limit hypertrophic scar tissue formation. They analyzed fibroblast and epithelial cell migration, collagen production and ROS activity, demonstrating the capability of the iron chelator to inhibit fibroblasts migration and their collagen production and to reduce pro-inflammatory cytokines release, such as IL-6 and also ROS production [84]. Moreover, DFP exerts anti-inflammatory properties also by inhibiting the formation of advanced glycation end products (AGEs) [85], known to directly stimulate inflammatory responses in innate immune cells [86].

### 3.2. Deferasirox

Deferasirox (DFX, Exjade, ICL670) is an FDA-approved iron chelating agent that offered better patient compliance to ICT compared with deferoxamine in patients with beta-thalassemia and sickle cell disease [87]. The iron overload in β-thalassemia causes OP [65,66]. In 2014 it has been demonstrated that DFX, through its iron chelating effects, plays a key role in counteracting OP in thalassemic patients by determining a decrease of OC activity [36]. Adel et al. investigated the potential antifibrotic effect of DFX in a model of liver fibrosis [88]. Liver fibrosis is characterized by an excess of extracellular matrix proteins produced by activated hepatic stellate cells (HSCs) which produce ROS and inflammatory mediators including TNF-α, interferon-γ (IFN-γ), and inducible nitric oxide synthase (iNOS), thus further increasing the fibrogenesis process [89]. DFX administration counteracts this inflammation by reducing inflammatory mediator levels and also by inhibiting NF-κB activation and HSCs proliferation. The DFX effect on NF-κB pathway has been demonstrated also by other authors. Meunier et al. demonstrated that DFX inhibits the expression of two target genes involved in the inflammatory response of NF-κB, interleukin 1 receptor type 1 (IL1R1) and Toll-like Receptor 4 (TLR4) [90].

Recent studies indicate that iron regulates Wnt signaling, and that the iron chelator DFX can inhibit Wnt signaling [91]. Wnt activation is known to enhance inflammatory processes [92], therefore it is implicated in the pathogenesis of several diseases [49]. The relation between iron excess and the alteration of Wnt signaling has been investigated in neurodegenerative disorders. Wu et al. investigated the ability of DFX in the treatment of abnormal Wnt signaling in a model of posthemorrhagic chronic hydrocephalus (PHCH), characterized by an increase of iron in cerebral spinal fluid (CSF) and of ferritin in the brain. They revealed that DFX, chelating iron in CSF and brain, normalizes the up-regulation of Wnt/β-catenin signaling, improving PHCH severity [93]. Recently, Nazari et al. confirmed the effects of iron chelators on adipose tissue, demonstrating that DFX is able to activate beige fat differentiation and metabolic activity, thus suggesting it as a potential therapeutic strategy to treat obesity and to prevent its metabolic complications. Interestingly, in C57Bl/6 mice, placed on high-fat diet and treated or not with DFX, they analyzed the inguinal fat, a classically brown or beige fat store in mice, observing an increase in UCP1 positive cells after DFX administration [94].

It is known that inflammatory processes are involved in cancer development, contributing to neoplastic cell transformation [95]. Interestingly, osteosarcoma (OS) progression is dependent on iron metabolism, indeed an alteration of this one is reported in OS. In recent years it has been demonstrated that DFX also plays a key role in counteracting OS progression, through its iron chelating properties [96].

### 3.3. Dp44mT

Di-2-pyridylketone-4,4-dimethyl-3-thiosemicarbazone (Dp44mT) is another important iron chelator, recently discovered to have anti-tumor properties resulting in a reduction of proliferation and an increase in apoptosis of several cancer cells [72]. It is known that cancer and inflammation are closely associated [97]. In particular, chronic inflammation is involved in cancer development, determining neoplastic cell transformation [95]. For this reason, targeting inflammation could be an innovative and useful strategy in order to counteract cancer progression and to prevent cancer development [97]. Nam et al. demonstrated that Dp44mT exerted anti-inflammatory properties on activated mast cells by modulating mainly NF-κB, MAPK, and hypoxia-inducible factor-1α (HIF-1α) pathways [72]. Dp44mT significantly induced both a strong reduction of pro-inflammatory cytokines release, among them IL-6, and also of HIF-1α activation. This interesting Dp44mT capability led the researchers to suggest it as anti-inflammatory molecule and, consequently, as anti-cancer drug, given the closely relation between cancer and inflammation [72]. Moreover, in 2017 it has been revealed that Dp44mT exerted anti-allergic inflammatory effects in both in vivo and in vitro models [72,98]. Recently, it has been reported an anti-inflammatory effect of Dp44mT also in severe COVID-19 infection, by modulating and containing the cytokine storm [99].

Interestingly, Lim and collaborators reported that Dp44mT had anti-inflammatory properties by modulating NF-kB pathways in macrophages activated with bacterial LPS [100]. Macrophages are the main cells involved in immune and inflammatory processes and also in iron metabolism [100,101]. When activated with LPS, they showed pro-inflammatory activities by producing several release factors involved in inflammation, among them IL-6, TNF-α, and NO, all involved in the onset of several inflammatory and immune diseases [100,102]. The administration of Dp44mT induced a reduction of these pro-inflammatory cytokines release in macrophage-mediated inflammatory processes by downregulating NF-κB activation, thus exerting anti-inflammatory effects [100].

## 4. Eltrombopag as an Anti-Inflammatory Molecule

Eltrombopag (ELT) is an orally available thrombopoietin receptor agonist responsible for the stimulation of platelet production, currently approved for the treatment of chronic immune thrombocytopenia when both first-line therapy and splenectomy fail [101,103,104]. It is also used in refractory aplastic anemia, and in patients with thrombocytopenia secondary to hepatitis C during treatment with interferon [104]. Although it has been demonstrated that ELT is effective in patients with myelodysplastic syndromes, it is not yet approved for this disease [104].

ELT is also known to be an immunomodulating drug, able to improve T and B regulatory cells activity and to inhibit T-cell responses to platelet auto-antigens [101,105]. It has also the capability to induce monocytes inactivation by determining a reversion of Fcγ receptors toward an inhibitory phenotype, reducing their phagocytic capacity [101,105,106].

In recent years, it has been proved that ELT is also able to bind the main intracellular iron form Fe^3+^, showing iron chelating properties [71,96,101,103,107,108]. In particular, in 2017 Vlachodimitropoulou et al. demonstrated in both human hepatoma cells (HuH7) and rat cardiomyocytes (H9C2) that ELT is a powerful iron chelator responsible for intracellular iron concentration reduction and iron mobilization when it was combined with other chelators, through a shuttling mechanism [108]. ELT administration resulted in a reduction of ROS and consequently in a decrease of ROS-related cell damage and an improvement of cell functions [108]. Effectively, the use of ELT as an iron chelator is encouraged due to its lipophilicity and low molecular weight which allow its cellular uptake and high chelator efficacy [108]. In the literature, the iron chelating properties of ELT are widely discussed. These properties seem to mediate the newly investigated anti-tumor properties of ELT. It is reported that it induces anti-proliferative and pro-apoptotic effects in leukemia cells by binding labile iron and reducing its intracellular concentration [109,110,111]. Argenziano et al. suggested a possible synergism between ELT and cytarabine in pediatric acute myeloid leukemia (AML) cell line (THP1), observing an increase of cancer cell apoptosis, a decrease of their viability and proliferation, and also an arrest of cell cycle progression when drugs were co-administered. These effects were probably due to iron chelating properties of ELT [107]. Anti-cancer effects of ELT mediated by its iron chelating properties are reported also in other cancers, among them Ewing sarcoma tumors [111], hepatocellular carcinoma [112].

**Figure 3:**
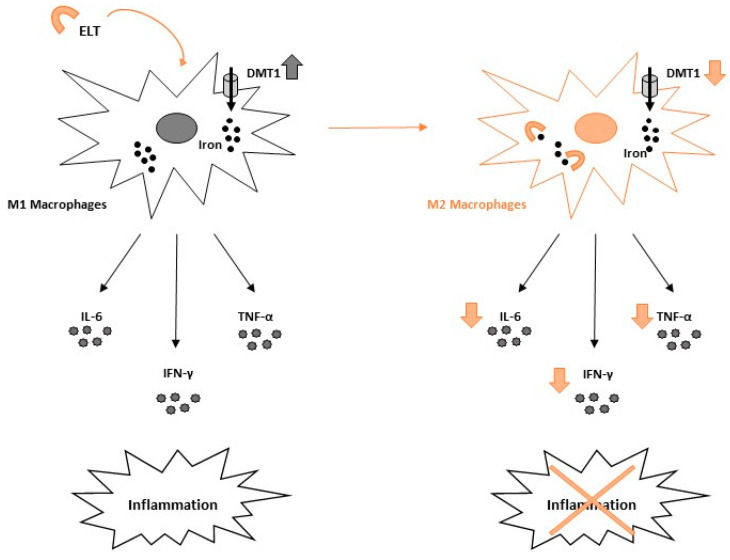
Eltrombopag effect on macrophages switch. Eltrombopag (ELT) administration induces a macrophage switch from the M1 pro-inflammatory phenotype towards the M2 anti-inflammatory one, by reducing intracellular iron concentration, divalent metal transporter 1 (DMT1) expression levels and pro-inflammatory cytokine release (Interleukin-6 (IL-6), tumor necrosis factor-α (TNF-α), and interferon-γ (IFN-γ)), thus counteracting inflammation.

Recently, it has been proven that ELT also exhibits interesting anti-inflammatory properties acting directly on cells involved in inflammation and indirectly by modulating iron metabolism. In 2020 it has been demonstrated that ELT administration induced a macrophage switch from M1 pro-inflammatory phenotype towards M2 anti-inflammatory one in immune thrombocytopenia (ITP), ameliorating the impaired inflammatory state of patients with ITP [101]. ELT was able to modulate directly inflammatory cytokine concentration by inducing both a reduction of pro-inflammatory cytokine levels and an increase of anti-inflammatory cytokine concentration, thus inducing macrophage polarization towards the M2 phenotype and consequently counteracting inflammation (Figure 3).

The anti-inflammatory effects of ELT were also investigated in mesenchymal stromal cells (MSCs) obtained from ITP patients [103]. In particular, it not only determined a reduction of inflammatory status by decreasing pro-inflammatory cytokine levels and increasing anti-inflammatory cytokine concentration, but also by acting on iron metabolism [103]. As discussed above, inflammation is responsible for iron accumulation in cells and, when in high concentration, it causes cell damage by determining ROS production [18,113]. IL-6 plays a key role in iron metabolism: it causes an increase of hepcidin concentration and activation, which induces FPN1 degradation and, consequently, iron release inhibition [18,114]. Interestingly, we already proposed another mechanism of action underlying ELT anti-inflammatory properties. They demonstrated that ELT administration not only reduced iron concentration in ITP MSCs through its iron chelating properties, but also by modulating the levels of both TfR-1 and FPN1 [103]. ELT induced a reduction of TfR-1 levels, inhibiting iron uptake by cells and an increase of FPN1 expression levels, allowing iron release from cells. In this way, ELT by modulating iron efflux is responsible for an intracellular iron concentration decrease, thus contributing to the amelioration of the inflammatory state, which is more compromised in ITP.

Moreover, it could be interesting to evaluate the effects of ELT also on ferroptosis, known to be associated with iron concentration and to be also involved in inflammation.

Considering iron involvement in inflammation and ELT iron chelating properties together with its interesting ability to modulate iron metabolism by reducing its concentration, the use of ELT can certainly be suggested and recommended to counteract the alteration of the characteristic inflammatory state of several immune and inflammatory diseases.

In conclusion, increasingly frequent off-label use of ELT as an anti-inflammatory and immunomodulatory drug is desirable in the future, given its already known iron chelating properties along with the recently discovered properties of modulating iron metabolism. The heterogeneity of iron-related inflammatory and autoimmune diseases and the use of glucocorticoids, commonly administered in these diseases but with several side effects, make necessary the identification of a novel and safety therapeutic strategy. Therefore, the use of ELT could be very promising in its off-label employment.

## 5. Conclusions

Iron is a crucial element for mammalian cells. It is involved in several vital mechanisms. In particular, it is the main constituent of several biomolecules essential for life, for example in the heme group of hemoglobin. Iron participates in cell growth, and proliferation [1].

Although it plays an important biologic role in cells, its excess causes the onset of several disorders [18]. Indeed, high levels of intracellular iron could be responsible for cell damage because it takes part in Fenton reactions, which catalyze the production of ROS. ROS accumulation induces lipid, protein, and nucleic acid injury, thus determining an alteration of cellular functions [5,6,7].

It is known that increased ROS production participates in inflammatory events, and also induces an increase of iron concentration, thus contributing to inflammation and establishing a vicious circle. Therefore, the maintenance of iron homeostasis equilibrium is of crucial importance for cells [5].

Several proteins participate in iron homeostasis, contributing to the regulation of intracellular iron concentration. TfR-1 and DMT1 are the main iron importers, responsible for iron intake in cells. FPN1 is the only known iron exporter involved in iron release by cells. Iron efflux is finely regulated both at cellular and systemic levels. At the cellular level, the iron regulatory proteins (IRPs), IRP1 and IRP2 are the main protagonists, participating in the modulation of DMT1, TfR-1, FPN1 and ferritin levels [15]. Instead, at the systemic level, hepcidin plays an important role. Hepcidin is a protein hormone responsible for FPN1 degradation, thus inhibiting iron release by cells and determining iron accumulation in cells [15,20].

Hepcidin activation is also dependent on inflammatory status. In particular, increased levels of pro-inflammatory cytokine IL-6 induce an increase of hepcidin levels and activation, resulting in a degradation of FPN1. In this way, cells cannot release iron. The accumulation of iron in cells causes cell damage and, consequently, the onset of several disorders. This mechanism is reported in several autoimmune and inflammatory diseases, so targeting iron and proteins involved in its metabolism regulation could be considered an innovative and useful strategy in order to better manage inflammation [18].

In the recent years, more attention is paid to iron chelators. In particular, it has been already reported an important role of DFO, DFP, DFX, and Dp44mTD as anti-inflammatory drugs [75,77,83,98]. These iron chelators are able to reduce pro-inflammatory cytokines release in several common inflammatory diseases, by both modulating NF-kB signaling pathway and chelating iron, known to be involved in inflammation.

Interestingly, ELT has recently been shown to play an important role as an anti-inflammatory molecule [101,103]. It is a thrombopoietin receptor agonist, approved for treatment of chronic ITP, responsible for stimulation of platelet production [105]. It has been demonstrated that it is also an immunomodulatory drug, involved in modulation of B and T regulatory cells and in inhibition of B cells. In recent years, more interest has been turned to its new emerging iron-chelating properties. It has been shown that ELT is able to modulate the impaired inflammatory state in different types of cancer and, in particular, in ITP [101,103]. It performs this task not only by acting directly on iron, chelating it and therefore reducing its intracellular concentration, but also indirectly by acting on the proteins responsible for modulation of its metabolism. Therefore, it could be very interesting to investigate these innovative anti-inflammatory effects of ELT in inflammatory and autoimmune diseases, highlighting its novel mechanism of action in regulation of inflammatory responses by modulation of iron metabolism. In this way, ELT could be proposed as a novel anti-inflammatory molecule.

Definitely, iron chelation seems to have an important role during inflammation thanks to its useful properties. Indeed, since iron accumulation leads to inflammation, the use of iron chelators could be suggested as a novel and interesting therapeutic strategy in order to counteract the impaired inflammatory state of several autoimmune and inflammatory diseases.

## Data Availability

Not applicable.

## Abbreviations

Tf	Transferrin
TfR-1	Transferrin Receptor-1
DMT1	Divalent Metal Transporter 1
FPN1	Ferroportin 1
IRPs	Iron regulatory proteins
IREs	Iron responsive elements
UTRs	Untranslated regions
mRNAs	messenger RNAs
LIP	Labile iron pool
ROS	Reactive oxygen species
TNF-α	Tumor necrosis factor-α
IL	Interleukin
NF-κB	Nuclear factor-kappa B
OP	Osteoporosis
OC	Osteoclast
OCs	Osteoclasts
ITP	Immune Thrombocytopenia
BMP	Bone morphogenetic protein
SMAD	Sma mothers against decapentaplegic
BMPRs	BMP receptors
BMP-RE	BMP-responsive elements
LIP	labile iron pool
ROS	Reactive oxygen species
IBD	Inflammatory Bowel Diseases
CD	Crohn’s disease
UC	ulcerative colitis
PUFAs	polyunsaturated fatty acids
GSH	glutathione
GPX4	glutathione peroxidase 4
VDACs	voltage-dependent anion channels
RANK-L	Nuclear factor-kappa B ligand
OPG	Osteoprotegerin
CVD	chronic venous disease
BM-MSCs	Bone Marrow Mesenchymal Stem Cells
DFO	deferoxamine
DFP	deferiprone
DFX	deferasirox
ELT	eltrombopag
TGF-β	tumor growth factor-β
AGEs	advanced glycation end products
IFN-γ	interferon-γ
iNOS	inducible nitric oxide synthase
IL1R1	interleukin 1 receptor type 1
TLR4	Toll-like Receptor 4
Dp44mT	Di-2-pyridylketone-4,4-dimethyl-3-thiosemicarbazone
PHCH	posthemorrhagic chronic hydrocephalus
CSF	cerebral spinal fluid
COVID-19	coronavirus disease 2019
LPS	lipopolysaccharide

## References

[1] Ni S., Yuan Y., Kuang Y., Li X. (2022). Iron Metabolism and Immune Regulation. Front. Immunol..

[2] Daher R., Manceau H., Karim Z. (2017). Iron metabolism and the role of the iron-regulating hormone hepcidin in health and disease. Presse Med..

[3] Cornelissen A., Guo L., Sakamoto A., Virmani R., Finn A.V. (2019). New insights into the role of iron in inflammation and atherosclerosis. Ebiomedicine.

[4] Kernan K.F., Carcillo J.A. (2017). Hyperferritinemia and inflammation. Int. Immunol..

[5] Anderson G.J., Frazer D.M. (2017). Current understanding of iron homeostasis. Am. J. Clin. Nutr..

[6] Vogt A.S., Arsiwala T., Mohsen M., Vogel M., Manolova V., Bachmann M.F. (2021). On Iron Metabolism and Its Regulation. Int. J. Mol. Sci..

[7] Gao G., Li J., Zhang Y., Chang Y.Z. (2019). Cellular Iron Metabolism and Regulation. Adv. Exp. Med. Biol..

[8] Brissot P., Ropert M., Le Lan C., Loreal O. (2012). Non-transferrin bound iron: A key role in iron overload and iron toxicity. Biochim. Biophys. Acta.

[9] Drakesmith H., Nemeth E., Ganz T. (2015). Ironing out Ferroportin. Cell Metab..

[10] Galaris D., Barbouti A., Pantopoulos K. (2019). Iron homeostasis and oxidative stress: An intimate relationship. Biochim. Biophys. Acta Mol. Cell Res..

[11] Nakamura T., Naguro I., Ichijo H. (2019). Iron homeostasis and iron-regulated ROS in cell death, senescence and human diseases. Biochim. Biophys. Acta Gen. Subj..

[12] Papanikolaou G., Pantopoulos K. (2005). Iron metabolism and toxicity. Toxicol. Appl. Pharm..

[13] Wilkinson N., Pantopoulos K. (2014). The IRP/IRE system in vivo: Insights from mouse models. Front. Pharmacol..

[14] Hawula Z.J., Wallace D.F., Subramaniam V.N., Rishi G. (2019). Therapeutic Advances in Regulating the Hepcidin/Ferroportin Axis. Pharmaceuticals.

[15] Nemeth E., Ganz T. (2021). Hepcidin-Ferroportin Interaction Controls Systemic Iron Homeostasis. Int. J. Mol. Sci..

[16] Martinelli M., Strisciuglio C., Alessandrella A., Rossi F., Auricchio R., Campostrini N., Girelli D., Nobili B., Staiano A., Perrotta S. (2016). Serum Hepcidin and Iron Absorption in Paediatric Inflammatory Bowel Disease. J. Crohns Colitis.

[17] Meynard D., Kautz L., Darnaud V., Canonne-Hergaux F., Coppin H., Roth M.P. (2009). Lack of the bone morphogenetic protein BMP6 induces massive iron overload. Nat. Genet..

[18] Camaschella C., Nai A., Silvestri L. (2020). Iron metabolism and iron disorders revisited in the hepcidin era. Haematologica.

[19] Ganz T., Nemeth E. (2009). Iron sequestration and anemia of inflammation. Semin. Hematol..

[20] Nemeth E., Rivera S., Gabayan V., Keller C., Taudorf S., Pedersen B.K., Ganz T. (2004). IL-6 mediates hypoferremia of inflammation by inducing the synthesis of the iron regulatory hormone hepcidin. J. Clin. Investig..

[21] Hussain T., Tan B., Yin Y., Blachier F., Tossou M.C., Rahu N. (2016). Oxidative Stress and Inflammation: What Polyphenols Can Do for Us?. Oxid. Med. Cell. Longev..

[22] Prabhakar O. (2013). Cerebroprotective effect of resveratrol through antioxidant and anti-inflammatory effects in diabetic rats. Naunyn Schmiedebergs Arch. Pharm..

[23] Wincup C., Sawford N., Rahman A. (2021). Pathological mechanisms of abnormal iron metabolism and mitochondrial dysfunction in systemic lupus erythematosus. Expert Rev. Clin. Immunol..

[24] Saleh J., Peyssonnaux C., Singh K.K., Edeas M. (2020). Mitochondria and microbiota dysfunction in COVID-19 pathogenesis. Mitochondrion.

[25] Paul B.T., Manz D.H., Torti F.M., Torti S.V. (2017). Mitochondria and Iron: Current questions. Expert Rev. Hematol..

[26] Aguirre J.D., Culotta V.C. (2012). Battles with iron: Manganese in oxidative stress protection. J. Biol. Chem..

[27] Rossi F., Tortora C., Argenziano M., Di Paola A., Punzo F. (2020). Cannabinoid Receptor Type 2: A Possible Target in SARS-CoV-2 (CoV-19) Infection?. Int. J. Mol. Sci..

[28] Edeas M., Saleh J., Peyssonnaux C. (2020). Iron: Innocent bystander or vicious culprit in COVID-19 pathogenesis?. Int. J. Infect. Dis..

[29] Kurz T., Eaton J.W., Brunk U.T. (2011). The role of lysosomes in iron metabolism and recycling. Int. J. Biochem. Cell Biol..

[30] Persson H.L., Richardson D.R. (2005). Iron-binding drugs targeted to lysosomes: A potential strategy to treat inflammatory lung disorders. Expert Opin. Investig. Drugs.

[31] Tortora C., Di Paola A., Creoli M., Argenziano M., Martinelli M., Miele E., Rossi F., Strisciuglio C. (2022). Effects of CB2 and TRPV1 Stimulation on Osteoclast Overactivity Induced by Iron in Pediatric Inflammatory Bowel Disease. Inflamm. Bowel Dis..

[32] Xu S., He Y., Lin L., Chen P., Chen M., Zhang S. (2021). The emerging role of ferroptosis in intestinal disease. Cell Death Dis..

[33] Chen Y., Zhang P., Chen W., Chen G. (2020). Ferroptosis mediated DSS-induced ulcerative colitis associated with Nrf2/HO-1 signaling pathway. Immunol. Lett..

[34] Xu M., Tao J., Yang Y., Tan S., Liu H., Jiang J., Zheng F., Wu B. (2020). Ferroptosis involves in intestinal epithelial cell death in ulcerative colitis. Cell Death Dis..

[35] Karaskova E., Volejnikova J., Holub D., Velganova-Veghova M., Sulovska L., Mihal V., Horvathova M., Pospisilova D. (2018). Hepcidin in newly diagnosed inflammatory bowel disease in children. J. Paediatr. Child Health.

[36] Rossi F., Perrotta S., Bellini G., Luongo L., Tortora C., Siniscalco D., Francese M., Torella M., Nobili B., Di Marzo V. (2014). Iron overload causes osteoporosis in thalassemia major patients through interaction with transient receptor potential vanilloid type 1 (TRPV1) channels. Haematologica.

[37] Sun Y., Chen P., Zhai B., Zhang M., Xiang Y., Fang J., Xu S., Gao Y., Chen X., Sui X. (2020). The emerging role of ferroptosis in inflammation. Biomed. Pharmacother..

[38] Dixon S.J., Lemberg K.M., Lamprecht M.R., Skouta R., Zaitsev E.M., Gleason C.E., Patel D.N., Bauer A.J., Cantley A.M., Yang W.S. (2012). Ferroptosis: An iron-dependent form of nonapoptotic cell death. Cell.

[39] Stockwell B.R., Friedmann Angeli J.P., Bayir H., Bush A.I., Conrad M., Dixon S.J., Fulda S., Gascon S., Hatzios S.K., Kagan V.E. (2017). Ferroptosis: A Regulated Cell Death Nexus Linking Metabolism, Redox Biology, and Disease. Cell.

[40] Yang W.S., SriRamaratnam R., Welsch M.E., Shimada K., Skouta R., Viswanathan V.S., Cheah J.H., Clemons P.A., Shamji A.F., Clish C.B. (2014). Regulation of ferroptotic cancer cell death by GPX4. Cell.

[41] Zhu G., Sui S., Shi F., Wang Q. (2022). Inhibition of USP14 suppresses ferroptosis and inflammation in LPS-induced goat mammary epithelial cells through ubiquitylating the IL-6 protein. Hereditas.

[42] Kim S., Keku T.O., Martin C., Galanko J., Woosley J.T., Schroeder J.C., Satia J.A., Halabi S., Sandler R.S. (2008). Circulating levels of inflammatory cytokines and risk of colorectal adenomas. Cancer Res..

[43] Guz-Mark A., Rinawi F., Egotubov O., Shimon I., Shamir R., Assa A. (2017). Pediatric-onset inflammatory bowel disease poses risk for low bone mineral density at early adulthood. Digest. Liver Dis..

[44] Semrin G., Fishman D.S., Bousvaros A., Zholudev A., Saunders A.C., Correia C.E., Nemeth E., Grand R.J., Weinstein D.A. (2006). Impaired intestinal iron absorption in Crohn’s disease correlates with disease activity and markers of inflammation. Inflamm. Bowel Dis..

[45] Tortora C., Di Paola A., Argenziano M., Creoli M., Marrapodi M.M., Cenni S., Tolone C., Rossi F., Strisciuglio C. (2022). Effects of CB2 Receptor Modulation on Macrophage Polarization in Pediatric Celiac Disease. Biomedicines.

[46] Trenam C.W., Blake D.R., Morris C.J. (1992). Skin inflammation: Reactive oxygen species and the role of iron. J. Investig. Dermatol..

[47] Wright J.A., Richards T., Srai S.K. (2014). The role of iron in the skin and cutaneous wound healing. Front. Pharmacol..

[48] Che J.M., Yang J.C., Zhao B., Zhang G., Wang L.Y., Peng S.L., Shang P. (2020). The Effect of Abnormal Iron Metabolism on Osteoporosis. Biol. Trace Elem. Res..

[49] Armstrong A., Mandala A., Malhotra M., Gnana-Prakasam J.P. (2022). Canonical Wnt Signaling in the Pathology of Iron Overload-Induced Oxidative Stress and Age-Related Diseases. Oxid. Med. Cell. Longev..

[50] Koskenkorva-Frank T.S., Weiss G., Koppenol W.H., Burckhardt S. (2013). The complex interplay of iron metabolism, reactive oxygen species, and reactive nitrogen species: Insights into the potential of various iron therapies to induce oxidative and nitrosative stress. Free. Radic. Biol. Med..

[51] Nakamura K., Kawakami T., Yamamoto N., Tomizawa M., Fujiwara T., Ishii T., Harigae H., Ogasawara K. (2016). Activation of the NLRP3 inflammasome by cellular labile iron. Exp. Hematol..

[52] Ruddell R.G., Hoang-Le D., Barwood J.M., Rutherford P.S., Piva T.J., Watters D.J., Santambrogio P., Arosio P., Ramm G.A. (2009). Ferritin functions as a proinflammatory cytokine via iron-independent protein kinase C zeta/nuclear factor kappaB-regulated signaling in rat hepatic stellate cells. Hepatology.

[53] Yu L.N., Wang S.J., Chen C., Rausch V., Elshaarawy O., Mueller S. (2021). Direct modulation of hepatocyte hepcidin signaling by iron. World J. Hepatol..

[54] Timmers P.R.H.J., Wilson J.F., Joshi P.K., Deelen J. (2020). Multivariate genomic scan implicates novel loci and haem metabolism in human ageing. Nat. Commun..

[55] Forrester S.J., Kikuchi D.S., Hernandes M.S., Xu Q., Griendling K.K. (2018). Reactive Oxygen Species in Metabolic and Inflammatory Signaling. Circ. Res..

[56] Chieppa M., Galleggiante V., Serino G., Massaro M., Santino A. (2017). Iron Chelators Dictate Immune Cells Inflammatory Ability: Potential Adjuvant Therapy for IBD. Curr. Pharm. Des..

[57] Borella E., Oosterholt S., Magni P., Della Pasqua O. (2022). Characterisation of individual ferritin response in patients receiving chelation therapy. Br. J. Clin. Pharmacol..

[58] Di Maggio R., Maggio A. (2017). The new era of chelation treatments: Effectiveness and safety of 10 different regimens for controlling iron overloading in thalassaemia major. Br. J. Haematol..

[59] Quarta A., Sgherza N., Pasanisi A., Solfrizzi M.P., Serra M., Vitucci A., Dello Iacono N., Renni R., Daprile C., Mosna F. (2020). Switching from dispersible to film coated tablet formulation of deferasirox improves hemoglobin levels and transfusional interval in patients with transfusion-dependent-thalassemia. Br. J. Haematol..

[60] Roberts D.J. (2017). Expanding access to Transfusion Medicine and improving practice: Guidelines, patient blood management, protocols and products. Transfus. Med..

[61] Botzenhardt S., Li N., Chan E.W., Sing C.W., Wong I.C., Neubert A. (2017). Safety profiles of iron chelators in young patients with haemoglobinopathies. Eur. J. Haematol..

[62] Maggio A., Kattamis A., Felisi M., Reggiardo G., El-Beshlawy A., Bejaoui M., Sherief L., Christou S., Cosmi C., Della Pasqua O. (2020). Evaluation of the efficacy and safety of deferiprone compared with deferasirox in paediatric patients with transfusion-dependent haemoglobinopathies (DEEP-2): A multicentre, randomised, open-label, non-inferiority, phase 3 trial. Lancet Haematol..

[63] Saliba A.N., El Rassi F., Taher A.T. (2016). Clinical monitoring and management of complications related to chelation therapy in patients with beta-thalassemia. Expert Rev. Hematol..

[64] Smith J.T., Schneider A.D., Katchko K.M., Yun C., Hsu E.L. (2017). Environmental Factors Impacting Bone-Relevant Chemokines. Front. Endocrinol.

[65] Danford C.J., Trivedi H.D., Bonder A. (2020). Bone Health in Patients With Liver Diseases. J. Clin. Densitom..

[66] Valenti L., Varenna M., Fracanzani A., Rossi V., Fargion S., Sinigaglia L. (2009). Association between iron overload and osteoporosis in patients with hereditary hemochromatosis. Osteoporos. Int..

[67] Katsumata S.I., Katsumata-Tsuboi R., Uehara M., Suzuki K. (2009). Severe Iron Deficiency Decreases Both Bone Formation and Bone Resorption in Rats. J. Nutr..

[68] Yang J.C., Meng X.F., Dong D.D., Xue Y.R., Chen X., Wang S.H., Shen Y., Zhang G.J., Shang P. (2018). Iron overload involved in the enhancement of unloading-induced bone loss by hypomagnetic field. Bone.

[69] Baschant U., Rauner M.T., Balaian E., Weidner H., Roetto A., Platzbecker U., Hofbauer L.C. (2016). Wnt5a is a key target for the pro-osteogenic effects of iron chelation on osteoblast progenitors. Haematologica.

[70] Fu X.R., Liu G., Halim A., Ju Y., Luo Q., Song G.B. (2019). Mesenchymal Stem Cell Migration and Tissue Repair. Cells.

[71] Punzo F., Tortora C., Argenziano M., Casale M., Perrotta S., Rossi F. (2018). Iron chelating properties of Eltrombopag: Investigating its role in thalassemia-induced osteoporosis. PLoS ONE.

[72] Nam S.Y., Han N.R., Yoon K.W., Kim H.M., Jeong H.J. (2017). Di-2-pyridylketone 4,4-dimethyl-3-thiosemicarbazone (Dp44mT), an anticancer agent, exerts an anti-inflammatory effect in activated human mast cells. Inflamm. Res..

[73] Guo Z., Lin J.M., Sun K., Guo J.Y., Yao X.D., Wang G.C., Hou L.C., Xu J.T., Guo J.C., Guo F.J. (2022). Deferoxamine Alleviates Osteoarthritis by Inhibiting Chondrocyte Ferroptosis and Activating the Nrf2 Pathway. Front. Pharmacol..

[74] Iwasaki T., Nakajima A., Yoneda M., Yamada Y., Mukasa K., Fujita K., Fujisawa N., Wada K., Terauchi Y. (2005). Serum ferritin is associated with visceral fat area and subcutaneous fat area. Diabetes Care.

[75] de Morais T.R., Gambero A. (2019). Iron chelators in obesity therapy—Old drugs from a new perspective?. Eur. J. Pharmacol..

[76] Gonzalez-Dominguez A., Visiedo-Garcia F.M., Dominguez-Riscart J., Gonzalez-Dominguez R., Mateos R.M., Lechuga-Sancho A.M. (2020). Iron Metabolism in Obesity and Metabolic Syndrome. Int. J. Mol. Sci..

[77] Wang S., Liu C., Pan S., Miao Q., Xue J., Xun J., Zhang Y., Gao Y., Duan X., Fan Y. (2015). Deferoxamine attenuates lipopolysaccharide-induced inflammatory responses and protects against endotoxic shock in mice. Biochem. Biophys. Res. Commun..

[78] Yan H.F., Liu Z.Y., Guan Z.A., Guo C. (2018). Deferoxamine ameliorates adipocyte dysfunction by modulating iron metabolism in ob/ob mice. Endocr. Connect..

[79] Wlazlo N., van Greevenbroek M.M.J., Ferreira I., Jansen E.H.J.M., Feskens E.J.M., van der Kallen C.J.H., Schalkwijk C.G., Bravenboer B., Stehouwer C.D.A. (2013). Iron Metabolism Is Associated With Adipocyte Insulin Resistance and Plasma Adiponectin The Cohort on Diabetes and Atherosclerosis Maastricht (CODAM) study. Diabetes Care.

[80] Hamaguchi K., Miyanishi K., Osuga T., Tanaka S., Ito R., Sakamoto H., Kubo T., Ohnuma H., Murase K., Takada K. (2022). Association between Hepatic Oxidative Stress Related Factors and Activation of Wnt/beta-Catenin Signaling in NAFLD-Induced Hepatocellular Carcinoma. Cancers.

[81] Xue H., Chen D., Zhong Y.K., Zhou Z.D., Fang S.X., Li M.Y., Guo C. (2016). Deferoxamine ameliorates hepatosteatosis via several mechanisms in ob/ob mice. Ann. N. Y. Acad. Sci..

[82] Mohammed A., Abd Al Haleem E.N., El-Bakly W.M., El-Demerdash E. (2016). Deferoxamine alleviates liver fibrosis induced by CCl_4_ in rats. Clin. Exp. Pharmacol. Physiol..

[83] Zou C., Liu X., Xie R., Bao Y., Jin Q., Jia X., Li L., Liu R. (2017). Deferiprone attenuates inflammation and myocardial fibrosis in diabetic cardiomyopathy rats. Biochem. Biophys. Res. Commun..

[84] Ramezanpour M., Smith J.L.P., Ooi M.L., Gouzos M., Psaltis A.J., Wormald P.J., Vreugde S. (2019). Deferiprone has anti-inflammatory properties and reduces fibroblast migration in vitro. Sci Rep..

[85] Sattarahmady N., Heli H., Moosavi-Movahedi A.A., Karimian K. (2014). Deferiprone: Structural and functional modulating agent of hemoglobin fructation. Mol. Biol. Rep..

[86] van der Lugt T., Weseler A.R., Gebbink W.A., Vrolijk M.F., Opperhuizen A., Bast A. (2018). Dietary Advanced Glycation Endproducts Induce an Inflammatory Response in Human Macrophages in Vitro. Nutrients.

[87] Meerpohl J.J., Schell L.K., Rucker G., Fleeman N., Motschall E., Niemeyer C.M., Bassler D. (2014). Deferasirox for managing iron overload in people with myelodysplastic syndrome. Cochrane Database Syst. Rev..

[88] Adel N., Mantawy E.M., El-Sherbiny D.A., El-Demerdash E. (2019). Iron chelation by deferasirox confers protection against concanavalin A-induced liver fibrosis: A mechanistic approach. Toxicol. Appl. Pharmacol..

[89] Bataller R., Brenner D.A. (2005). Liver fibrosis. J. Clin. Investig..

[90] Meunier M., Ancelet S., Lefebvre C., Arnaud J., Garrel C., Pezet M., Wang Y., Faure P., Szymanski G., Duployez N. (2017). Reactive oxygen species levels control NF-kappaB activation by low dose deferasirox in erythroid progenitors of low risk myelodysplastic syndromes. Oncotarget.

[91] Ziaei A., Ardakani M.R.P., Hashemi M.S., Peymani M., Ghaedi K., Baharvand H., Nasr-Esfahani M.H. (2015). Acute course of deferoxamine promoted neuronal differentiation of neural progenitor cells through suppression of Wnt/beta-catenin pathway: A novel efficient protocol for neuronal differentiation. Neurosci. Lett..

[92] Jridi I., Cante-Barrett K., Pike-Overzet K., Staal F.J.T. (2021). Inflammation and Wnt Signaling: Target for Immunomodulatory Therapy?. Front. Cell Dev. Biol..

[93] Wu J.M., Hua Y., Keep R.F., Nakamura T., Hoff J.T., Xi G.H. (2003). Iron and iron-handling proteins in the brain after intracerebral hemorrhage. Stroke.

[94] Nazari M., Ho K.W., Langley N., Cha K.M., Kodsi R., Wang M., Laybutt D.R., Cheng K., Stokes R.A., Swarbrick M.M. (2022). Iron chelation increases beige fat differentiation and metabolic activity, preventing and treating obesity. Sci. Rep..

[95] Khandia R., Munjal A. (2020). Interplay between inflammation and cancer. Adv. Protein Chem. Struct. Biol..

[96] Argenziano M., Di Paola A., Tortora C., Di Pinto D., Pota E., Di Martino M., Perrotta S., Rossi F., Punzo F. (2021). Effects of Iron Chelation in Osteosarcoma. Curr. Cancer Drug Targets.

[97] Singh N., Baby D., Rajguru J.P., Patil P.B., Thakkannavar S.S., Pujari V.B. (2019). Inflammation and cancer. Ann. Afr. Med..

[98] Kim H.Y., Han N.R., Kim H.M., Jeong H.J. (2018). The Iron Chelator and Anticancer Agent Dp44mT Relieves Allergic Inflammation in Mice With Allergic Rhinitis. Inflammation.

[99] Zabetakis I., Lordan R., Norton C., Tsoupras A. (2020). COVID-19: The Inflammation Link and the Role of Nutrition in Potential Mitigation. Nutrients.

[100] Lim J.H., Kim H.Y., Lee J.S., Kim H.M., Jeong H.J. (2021). Dp44mT regulates the levels of inflammatory mediators through blocking NF-kappaB nuclear translocation in LPS-stimulated RAW 264.7 macrophages. Vitr. Cell. Dev. Biology. Anim..

[101] Di Paola A., Palumbo G., Merli P., Argenziano M., Tortora C., Strocchio L., Roberti D., Santoro C., Perrotta S., Rossi F. (2020). Effects of Eltrombopag on In Vitro Macrophage Polarization in Pediatric Immune Thrombocytopenia. Int. J. Mol. Sci..

[102] Reddy D.B., Reddanna P. (2009). Chebulagic acid (CA) attenuates LPS-induced inflammation by suppressing NF-kappa B and MAPK activation in RAW 264.7 macrophages. Biochem. Biophys. Res. Commun..

[103] Di Paola A., Palumbo G., Tortora C., Argenziano M., Catanoso M., Di Leva C., Ceglie G., Perrotta S., Locatelli F., Rossi F. (2022). Eltrombopag in paediatric immune thrombocytopenia: Iron metabolism modulation in mesenchymal stromal cells. Br. J. Haematol..

[104] Fattizzo B., Levati G., Cassin R., Barcellini W. (2019). Eltrombopag in Immune Thrombocytopenia, Aplastic Anemia, and Myelodysplastic Syndrome: From Megakaryopoiesis to Immunomodulation. Drugs.

[105] Bortolotti M., Pettine L., Zaninoni A., Croci G.A., Barcellini W., Fattizzo B. (2022). Efficacy and Immunomodulating Properties of Eltrombopag in Aplastic Anemia following Autologous Stem Cell Transplant: Case Report and Review of the Literature. Pharmaceuticals.

[106] Liu X.G., Liu S., Feng Q., Liu X.N., Li G.S., Sheng Z., Chen P., Liu Y., Wei Y., Dong X.Y. (2016). Thrombopoietin receptor agonists shift the balance of Fcgamma receptors toward inhibitory receptor IIb on monocytes in ITP. Blood.

[107] Argenziano M., Tortora C., Paola A.D., Pota E., Martino M.D., Pinto D.D., Leva C.D., Rossi F. (2021). Eltrombopag and its iron chelating properties in pediatric acute myeloid leukemia. Oncotarget.

[108] Vlachodimitropoulou E., Chen Y.L., Garbowski M., Koonyosying P., Psaila B., Sola-Visner M., Cooper N., Hider R., Porter J. (2017). Eltrombopag: A powerful chelator of cellular or extracellular iron(III) alone or combined with a second chelator. Blood.

[109] Kalota A., Selak M.A., Garcia-Cid L.A., Carroll M. (2015). Eltrombopag modulates reactive oxygen species and decreases acute myeloid leukemia cell survival. PLoS ONE.

[110] Roth M., Will B., Simkin G., Narayanagari S., Barreyro L., Bartholdy B., Tamari R., Mitsiades C.S., Verma A., Steidl U. (2012). Eltrombopag inhibits the proliferation of leukemia cells via reduction of intracellular iron and induction of differentiation. Blood.

[111] Waters T., Goss K.L., Koppenhafer S.L., Terry W.W., Gordon D.J. (2020). Eltrombopag inhibits the proliferation of Ewing sarcoma cells via iron chelation and impaired DNA replication. BMC Cancer.

[112] Kurokawa T., Murata S., Zheng Y.W., Iwasaki K., Kohno K., Fukunaga K., Ohkohchi N. (2015). The Eltrombopag antitumor effect on hepatocellular carcinoma. Int. J. Oncol..

[113] Ginzburg Y.Z. (2019). Hepcidin-ferroportin axis in health and disease. Vitam. Horm..

[114] Mehta K.J. (2021). Role of iron and iron-related proteins in mesenchymal stem cells: Cellular and clinical aspects. J. Cell. Physiol..

